# Metabolic Abnormalities in Patients Prescribed Antipsychotics for Severe Mental Illness in the community

**DOI:** 10.1192/bjo.2025.10152

**Published:** 2025-06-20

**Authors:** Qammar Jabbar, Ravindra Belgamwar, Jason McDonald

**Affiliations:** North Staffordshire Combined Healthcare NHS Trust, Stoke-on-Trent, United Kingdom

## Abstract

**Aims:** To determine the prevalence of metabolic abnormalities in patients with severe mental health illness (SMI) prescribed antipsychotics by the local Community Mental Health Team at the North Staffordshire Combined Healthcare NHS Trust.

**Methods:** This cross-sectional study collected data for patients who were on antipsychotic medications in 2022. The variables assessed include age, sex, smoking status, ethnicity, substance use, body mass index (BMI), prolactin, HBA1c, HDL, LDL, non-HDL, and total cholesterol. The statistical analyses were performed using SPSS version 26.

**Results:** A total of 296 patients (mean age 51.4 years), 161 (54.4%) males and 135 (45.6%) females were included in the study. Most of the patients (91.5%) were white, whereas others belonged to Asian (2.36%), Black (1%), other ethnicities(2.02%), and 3.04% did not state their backgrounds. 112 (37.8%) were smokers, 86 (29%) were ex-smokers, and 90 (30.4%) were non-smokers. More than two-thirds, 256 (86.4%) of the patients had no history of substance use, but the remaining 40 (13.5%) were using or had a history of substance use. More than two-thirds (80.9%) of the patients were overweight, obese, or severely obese. 18.2% and 0.67% of the patients were normal or underweight, respectively. The HbA1c was increased in 85 (28.7%) and was in the normal range and not documented in 207 (69.9%) and 4 (1.35%) of the patients. Prolactin was found to be in the normal range in 192 (64.86%) cases but out of the normal range in more than a third of patients, 101 (34.12%), and was not reported in 3 (1.01%) cases. LDL was found to be within range in 224 (75.6%) of the patients, out of range in 46 (15.5%), and not recorded in 26 (8.7%) cases. Furthermore, HDL and total cholesterol were found within the normal values for 214 (72.3%) and 197 (66.55) of the patients, out of range for 77 (26.01%) and 95 (32.09%) cases and not documented for 5 (1.68%) and 4 (1.35%) of patients, respectively.

**Conclusion:** Patients on antipsychotic medications have a higher prevalence of metabolic abnormalities, such as increased risk of obesity, diabetes, and deranged cholesterol; however, the variation in metabolic profile in patients on different antipsychotics needs to be further explored. It is recommended that these patients be monitored at recommended intervals in accordance with the NICE guidelines for early detection and intervention of metabolic abnormalities to improve quality of life.

